# The Antiaging Activity of Ergothioneine in UVA-Irradiated Human Dermal Fibroblasts via the Inhibition of the AP-1 Pathway and the Activation of Nrf2-Mediated Antioxidant Genes

**DOI:** 10.1155/2020/2576823

**Published:** 2020-02-12

**Authors:** You-Cheng Hseu, Yugandhar Vudhya Gowrisankar, Xuan-Zao Chen, Yi-Chen Yang, Hsin-Ling Yang

**Affiliations:** ^1^Department of Cosmeceutics, College of Biopharmaceutical and Food Sciences, China Medical University, Taichung 40402, Taiwan; ^2^Department of Health and Nutrition Biotechnology, Asia University, Taichung 41354, Taiwan; ^3^Chinese Medicine Research Center, China Medical University, Taichung 40402, Taiwan; ^4^Research Center of Chinese Herbal Medicine, China Medical University, Taichung 40402, Taiwan; ^5^Institute of Nutrition, College of Biopharmaceutical and Food Sciences, China Medical University, Taichung 40402, Taiwan

## Abstract

UVA irradiation induced ROS-mediated photo damage to the human skin leading to coarseness, wrinkling, pigmentation, and cutaneous malignancies. We investigated the dermatoprotective efficacies of submicromolar concentrations of ergothioneine (EGT, 0.125-0.5 *μ*M), which occurs naturally as a sulfur-containing amino acid, in the mechanisms in human skin fibroblast (HSF) cells. UVA-induced AP-1 (c-Fos and c-Jun) translocation was found to be inhibited by EGT treatments with the parallel inhibition of the collagenolytic matrix metalloproteinase- (MMP-) 1 activation and type I procollagen degradation. Moreover, EGT mitigated UVA-induced ROS generation. An increase in the amount of antioxidant genes (HO-1, NQO-1, and *γ*-GCLC) from EGT and were associated with upregulated Nrf2 expressions in a dose-dependent or time-dependent manner. We confirmed this from Nrf2 translocation and increased nuclear ARE promoter activity that underlie EGT dermatoprotective activities. Also, glutathione (GSH) levels (from *γ*-GCLC) were significantly increased. Moreover, we showed that mediated by ERK, JNK, and PKC, signaling cascades mediate Nrf2 translocation. We confirmed this phenomenon by the suppressed nuclear Nrf2 activation in cells that were treated with respective inhibitors (PD98059, SP600125, and GF109203X). However, antioxidant protein expressions were impaired in Nrf2 knockdown cells to confirm that ARE/Nrf2 pathways and the inhibition of AP-1 had significant roles in EGT-mediated protective effects. We can conclude that ergothioneine ameliorated UVA-induced skin aging and is a useful food supplement for skin care products.

## 1. Introduction

The aging of skin is not a simple biological process, and it includes a combination of intrinsic and extrinsic factors to change functional and structural aspects of the skin. Growing evidence indicates that they have converging biochemical and molecular pathways. Photoaged skin is characterized by coarseness, wrinkling, sallow color, telangiectasia, depigmentation, and cutaneous malignancies. Photo damage is can be seen by disorganized collagen and the accumulation of tangles, amorphous, elastin-containing material (solar elastosis) [1–3]. The photoaging process is caused by changes in gene expressions in fibroblasts apparent in the dermal compartment [4, 5].

The UV light (290-400 nm) coming from the sun is composed of UVC (200-290 nm), UVB (290-320 nm), and UVA (320-400 nm). The ozone absorbs 100% of UVC, 90% of UVB, and no UVA. This UVA penetrates deeper and can also interact with fibroblasts [6, 7]. Fibroblasts primarily produce cells that generate collagen in the dermis. Members of matrix metalloproteases (MMPs) mediate the turnover of collagen. UVA could also alter DNA through the formation of reactive oxygen species (ROS) like peroxide, superoxide anion, and singlet oxygen [8, 9]. ROS assumes a basic priority in the aging of skin as well as photoaging. ROS activates MAP kinase signaling pathways, which further result in nuclear factor-*κ*B (NF-*κ*B) and with the activation of AP-1. AP-1 stimulates transcription of MMPs in fibroblasts and keratinocytes. It also inhibits type-1 procollagen generation in fibroblast cells [4, 6, 10, 11]. MMPs degrade the extracellular matrix and include MMP-1 (collagenase) which cleavages collagen type 1 and type III, MMP-3 (stromelysin), and MMP-9 (gelatinase) which further degrade the cleaved subunits [12]. NF-*κ*B stimulates transcription of inflammatory cytokines which also increase ROS levels [13, 14]. The skin is equipped with antioxidants that scavenge free radicals [15]. Various endogenous plant-derived and chemical antioxidants have been tested [16]. Most antioxidants are used topically because oral intake does not achieve elevated levels in the skin [17, 18].

L-Ergothioneine (EGT, 2-mercaptohistidine trimethyl betaine) is an amino acid that occurs naturally. It is a thiourea derivative of histidine that has a sulfur atom on its imidazole ring. EGT is produced by few organisms. The organs that produce it are actinobacteria, cyanobacteria, and certain fungi, among others [19]. EGT is produced almost exclusively from diet in humans. It also accumulates in parts of the human body such as the bone marrow, liver, erythrocytes, kidney, seminal fluids, eyes, and skin [20, 21]. It is found in bacteria, animals, and plants. Foods that contain EGT are the liver, kidney, black beans, oat bran, and kidney beans. The highest levels of EGT are in bolete and oyster mushrooms [22]. Its *in vivo* effects are under current research; the role it plays in humans is undetermined physiologically [20]. Bazela et al. in 2014 indicated that EGT is able to enhance levels of glutathione. They also indicated that it protected cells from photoaging-associated mitochondrial DNA. This also known as “common deletion” and is a phenomenon that occurs in skin cells. The authors of that study have suggested that EGT is effective for skin care and can be used effectively as an antiphotoaging ingredient [23]. The literature rarely mentioned EGT for direct application to the skin for wrinkle prevention. Additionally, it can also reduce signs of aging skin and damage from the sun. Moreover, treatments that use EGT have been shown to scavenge ^1^O_2_ and O_2_^−^ radicals. It can also suppress the MMP-1 expression in UV-irradiated dermal fibroblasts [24]. However, its inhibitory effects and molecular mechanisms are not well defined in human skin cells. As such, this study demonstrates the protective mechanisms exhibited by EGT against UVA-irradiated ROS-mediated collagen degradation, AP-1 signaling, and photodermato toxicity effects in human skin fibroblast (HSF) cells. This study also delineates the related molecular pathways that underlie the aforementioned effects.

## 2. Materials and Methods

### 2.1. Reagents and Antibodies

Fetal bovine serum (FBS), glutamine, Dulbecco's modified Eagle medium/high glucose (DMEM/HG), and penicillin/streptomycin were acquired from Gibco BRL (Grand Island, NY, USA). Ergothioneine (EGT) was procured from Sigma-Aldrich (St. Louis, MO). 3-[4,5-Dimethyl-2-yl]-2,5-diphenyl tetrazolium bromide (MTT), 2′,7′-dichlorofluorescin-diacetate (DCFH_2_-DA), PI3K/AKT inhibitor (LY294002), and antibodies for MMP-1, type I procollagen, and mouse monoclonal IL1*β* were obtained from Abcam Inc. (Cambridge, UK). Antibodies against p-c-Fos, p-c-Jun, Nrf2, NQO-1, ICAM-1, and *β*-actin were purchased from Santa Cruz Biotechnology Inc. (Heidelberg, Germany). Histone was purchased from Cell Signaling Technology (Beverly, MA, USA). Anti-HO-1 and anti-*γ*-GCLC antibodies were acquired from GeneTex Inc. (San Antonio, TX, USA). Pharmacological inhibitors of ERK (PD98059), JNK (SP600125), p38 MAPK (SB203580), and PKC (GF109203X) were obtained from Calbiochem (La Jolla, CA). All other chemicals and lab-ware were of the best quality that is commercially available and were acquired from either Merck & Co., Inc. (Darmstadt, Germany) or Sigma-Aldrich.

### 2.2. Cell Culture

Human dermal fibroblast (HSF) cell line was obtained from the BCRC (Food Industry Research and Development Institute, Hsinchu, Taiwan). Cells were maintained in a humidified atmosphere (95% air and 5% CO_2_ at 37°C) and were promulgated in the DMEM supplemented with 10% heat-inactivated FBS, 2 mM glutamine, and 1% penicillin/streptomycin. Cultures were harvested and cell morphology was examined using phase contrast microscopy. Cell number was monitored by counting cell suspensions using a hemocytometer (Marienfeld, Germany).

### 2.3. UVA Irradiation and Sample Treatment

Before UVA irradiation, we pretreated all cells with different concentrations of EGT (0.125-0.5 *μ*M) or vehicle (0.1% DMSO) for 24 h. Prior to incubation, PBS-washed cells were then resuspended in new phenol red-free DMEM containing 10% FBS. We further irradiated these cells with 3 J/cm^2^ (for 27 min) of UVA (*λ*_max_, 365 nm; no detectable emission below 320 nm) using the UVILink CL-508L (UVItec, Cambridge, UK). After this incubation period, cells were fixed and or harvested to perform subsequent experiments in this study. We used 50 *μ*M of EGT solution prepared in PBS as our stock solution. It was also stored at -20°C.

### 2.4. MTT Assay

We measured cell viability with an MTT colorimetric assay. We preincubated HSF cells (1 × 10^5^ cells/well in a 12-well plate) in the presence or absence of EGT. This was then followed by irradiation along with UVA. After this, these irradiated and PBS washed cells were incubated with 400 *μ*L of 0.5 mg/mL MTT in PBS for 2 h. We removed the culture supernatant and then we resuspended it with 400 *μ*L of isopropanol to dissolve MTT formazan. We also measured the absorbance of color developed at 570 nm with an ELISA microplate reader (BioTek Instruments, Winooski, VT, USA). Data were represented by the percentage of EGT exposed and viable cells that were then compared to the control cells and randomly assigned a viability value of 100%.

### 2.5. Preparation of Cytosolic and Nuclear Extracts

HSF cells (1 × 10^6^ cells/dish) were pretreated with EGT for the indicated time followed by exposure to UVA irradiation. After treatments, PBS-washed cells were allowed to incubate in ice for 15 min in the presence of lysis buffer (10 mM HEPES (pH 8.0), 0.1 mM EDTA, 10 mM KCl, 100 *μ*M EGTA, 1 mM DTT, 500 *μ*M PMSF, 2.0 *μ*g/mL leupeptin, 2.0 *μ*g/mL aprotinin, and 500 *μ*g/mL benzamidine). Finally, 10% (*v*/*v*) NP-40 (15 *μ*L) was put in with the cell lysate. These samples were then centrifuged (12000 × *g* for 5 min), and all collected supernatants were given as cytosolic protein extract. We resuspended the nuclear pellet in cold extraction buffer (20 mM HEPES (pH 8.0), 1 mM EDTA, 400 mM NaCl, 1 mM EGTA, 1 mM DTT, 1 mM PMSF, 2.0 *μ*g/mL leupeptin, 20 *μ*g/mL aprotinin, and 500 *μ*g/mL benzamidine). We then incubated it for 15 min. Nuclear protein extract was clarified at 15000 × *g* for 30 min. The concentrations of both cytoplasmic and nuclear proteins were determined by Bio-Rad protein assay method as prescribed by the company protocol, and the samples were preserved at −80°C.

### 2.6. Western Blot

We resolved equal concentrations of denatured proteins on 8–15% SDS-PAGE polyacrylamide gradient gel. We transferred the separated proteins onto polyvinylidene difluoride membranes. To avoid the nonspecific binding, thee PVDF membranes were gated with blotto (5% nonfat dried milk in PBS containing 1% Tween-20) for 1 h at room temperature and then incubated overnight with different primary antibodies at 4°C. The next day, primary antibodies were retained, then the membranes were washed and reincubated with either horseradish peroxidase-conjugated goat anti-rabbit or anti-mouse antibodies for 2 h at room temperature. Using the SuperSignal West Pico chemiluminescence substrate (Thermo Scientific Inc., Rockford, IL, USA), the immunoreactive protein bands were visualized and the images were captured by an ImageQuant™ LAS 4000 mini (Fujifilm) system.

### 2.7. Measurement of ROS Generation

The UVA irradiation-induced intracellular ROS accumulation was measured by the DCFH2-DA dye method. Approximately 0.1 million cells were allowed to grow to reach 80% confluence. We pretreated these cells with different concentrations of EGT for the indicated time. They were also irradiated with UVA. After the treatments, we incubated PBS-washed cells with 10 *μ*M DCFH2-DA in a new culture medium at 37°C for 30 min. Intracellular esterases acted upon the DCFH2-DA dye to remove the acetate groups and trap the probe inside the HSF cells. From the DCF fluorescence, we measured intracellular ROS with a fluorescence microscope (Olympus 1 × 71 at 200x magnification). We quantified the fluorescence intensity under each condition from a squared section of fluorescent-stained cells by analySIS LS 5.0 Soft Imaging Solutions (Olympus Imaging America Inc., Corporate Parkway Centre Valley, PA, USA).

### 2.8. Immunofluorescence

We seeded HSF cells (2 × 10^4^ cells/well) in an 8-well glass Lab-Tek chamber. We also pretreated them with different concentrations of EGT and then exposed them to UVA. After treatments, we fixed all PBS-washed cells with 4% paraformaldehyde (for 15 min) and permeabilized with 0.1% Triton X-100 (for 10 min). Then, we blocked them with 10% FBS in PBS. We incubated these cells with anti-p-c-Fos and anti-p-c-Jun primary antibodies in 1.5% FBS for overnight. The next day, we incubated the cells with FITC- (fluorescein isothiocyanate-) conjugated (488 nm) secondary antibodies for an additional 1 h in 6% bovine serum albumin. We counter-stained the cells with 1 *μ*g/mL DAPI for 5 min, and they were then visualized with a fluorescence microscope at ×200 magnification.

### 2.9. ARE-Transcriptional Activity/Luciferase Reporter Assay

We used a dual-luciferase reporter assay system (Promega, Madison, WI, USA) to measure the ARE-transcriptional activity assay. HSF cells (5 × 10^4^ cells/well) that have reached an ~80% confluence were made quiescent by incubating the cells in serum and antibiotic-free medium for 5 h. Using the Lipofectamine 2000 (Invitrogen, Carlsbad, CA, USA), we transfected the cells with either an ARE plasmid or a pcDNA vector with *β*-galactosidase. We treated the transfected cells with various concentrations of EGT and then they were exposed to UVA. We quantified the intensity of relative luminescence with a luminometer (BioTek Instruments Inc., Winooski, VA, USA). We normalized ARE luciferase activity to *β*-galactosidase activity in cell lysates.

### 2.10. Determination of Intercellular Glutathione (GSH)

We pretreated HSF cells (1 × 10^5^ cells) with different concentrations of EGT and then exposed them to UVA irradiation. We quantified the total GSH content in the culture media with a GSH assay kit (Cayman Chemical Co., Ann Arbor, MI). We used the kit works for an enzymatic recycling method, which involved the glutathione reductase enzyme to quantify the GSH levels. We measured the GSH content present in the treated cells by extrapolating the absorbance values of treated samples from the constructed standard curve.

### 2.11. siRNA Transfection in HSF Cells

We performed siRNA transfection in HSF cells with the Lipofectamine RNAiMAX (Invitrogen, Carlsbad, CA, USA) kit in accordance with the manufacturer's instructions. We seeded 5 × 10^5^ HSF cells/well in a 6-well plate and allowed to them to reach a 40–60% confluence, then we performed the siRNA transfection using the Lipofectamine RNAiMAX (Invitrogen, Carlsbad, CA, USA) kit. We used the same procedure for this study as in our previous studies (FRBM, 86, 2015, pg. 102–117). Transfected cells were treated with various concentrations of EGT followed by exposure to the UVA irradiation, and the intracellular ROS generation as well as the protein expression patterns was measured.

### 2.12. Statistical Analysis

We present our results as the mean ± standard deviation (mean ± SD). We analyzed all data by an analysis of variance, and then we followed that with Dunnett's test for pairwise comparisons. We defined statistical significance as ^∗^*p* < 0.05, ^∗∗^*p* < 0.01, and ^∗∗∗^*p* < 0.001 when compared to untreated control cells and ^#^*p* < 0.05, ^##^*p* < 0.01, and ^###^*p* < 0.001 when compared to UVA-irradiated cells.

## 3. Results

### 3.1. EGT Pretreatment Suppressed MMP-1 Expression but Increased the Type 1 Procollagen Expression in UVA-Exposed HSF Cells

**Figure 1:**
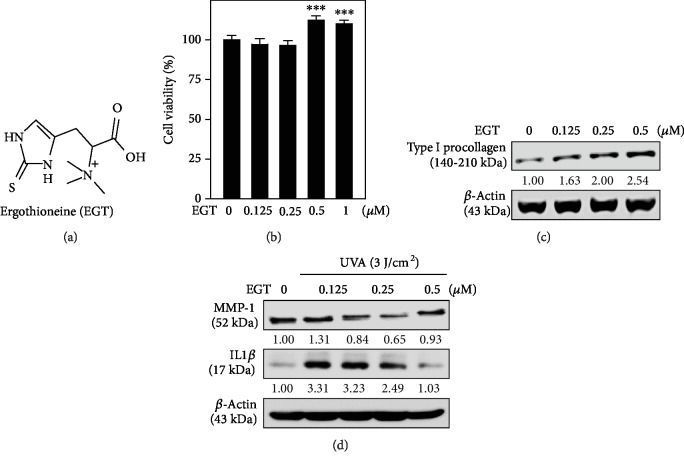
EGT suppressed MMP-1 and IL1*β* expression but enhanced the procollagen expression in HSF cells. (a) Chemical structure of ergothioneine (EGT). (b, c) Different concentrations of EGT (0.125-1 *μ*M) or vehicle (PBS) were treated with HSF cells for 24 h. (b) The percentage of cell viability was measured by the MTT colorimetric method. The formula used to calculate the percentage of viable cells was (*A*_570_ of treated cells/*A*_570_ of untreated cells) × 100. (c) EGT-mediated type I procollagen expression was measured by Western blot method. (d) HSF cells were pretreated with EGT (0.125-0.5 *μ*M) for 24 h and then irradiated without or with 3 J/cm^2^ UVA. The expression of MMP-1, IL1*β* proteins were measured by Western blot method against *β*-active as the internal control. Results were presented as mean ± SD of three or more assays. ^∗∗∗^*p* < 0.001 compared with untreated control cells.

We first tested the effect of EGT (Figure 1(a)) concentration on the viability of HSF cells. Our MTT data showed that EGT treatments at 0.5 and 1 *μ*M concentrations significantly upregulated the percentage of HSF cell viability compared to that of the untreated control cells (Figure 1(b)). There was only a negligible decrease in cell viability observed at lower EGT concentrations.

Later, the dermatoprotective properties of EGT in HSF cells were demonstrated. It is a well-known fact that UVA radiation-induced premature skin aging was associated with MMP-1 activation and collagen degradation events [25, 26]. Therefore, the effects of EGT concentrations on type I procollagen levels were tested. Our Western blot data showed that EGT dose-dependently increased the expression of type I procollagen expression, thus protecting the HSF cells from UVA radiation-induced collagen degradation (Figure 1(c)). On the other hand, we tested the effect of EGT pretreatment on the expression patterns of MMP-1 and IL1*β* proteins in UVA-irradiated (3 J/cm^2^) HSF cells.  Figure 1(d) shows that when compared to the UVA alone exposed cells, HSF cells pretreated with EGT followed by exposure to UVA showed dose-dependent decrease in the expression of the IL1*β* protein. But this pattern was observed up to 0.25 *μ*M EGT concentration only. All this data gave us an idea that EGT not only increases the viability of HSF cells but also protects these cells by reducing the MMP-1 degradation and enhancing the type I collagen production.

### 3.2. EGT Pretreatment Suppressed the UVA-Induced AP-1 Activation in HSF Cells

**Figure 2:**
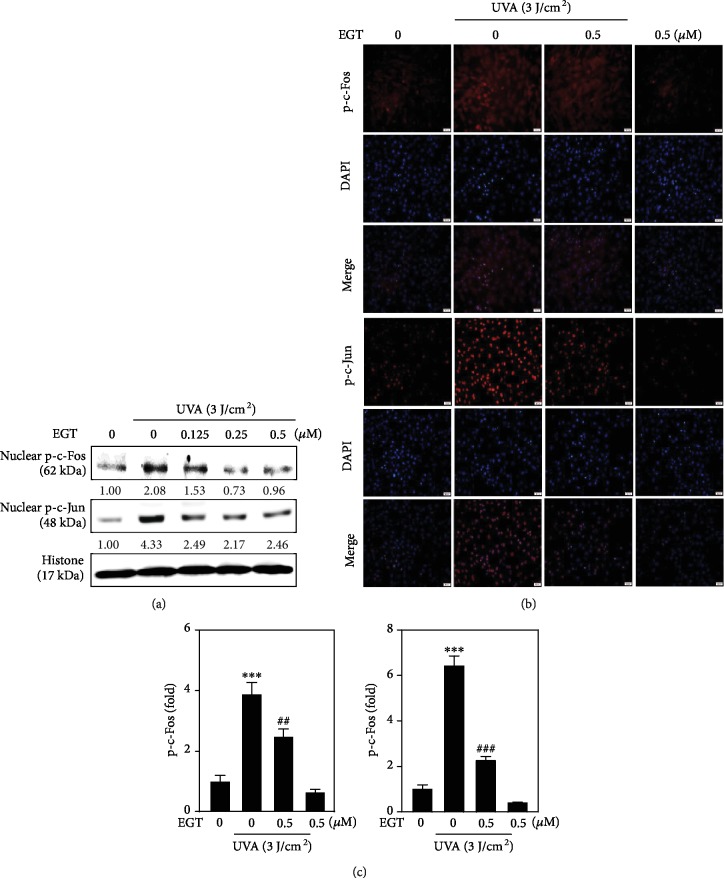
Inhibition of AP-1 activation by EGT pretreatment in UVA-irradiated HSF cells. HSF cells were pretreated with different concentrations of EGT (0.125-0.5 *μ*M) for 24 h followed by irradiated with 3 J/cm^2^ UVA. (a) EGT pretreatment suppressed the UVA-irradiated transactivation of nuclear c-Fos and c-Jun proteins in HSF cells. Western blot analysis of nuclear expression of phosphorylated c-Fos and c-Jun proteins was tested using their corresponding antibodies. Histone protein was used as an internal control. (b, c) Immunofluorescence staining indicates changes in AP-1 (p-c-Fos and p-c-Jun) expression. EGT pretreatment suppressed UVA-irradiated nuclear translocation of p-c-Fos and p-c-Jun in UVA-irradiated HSF cells. The percentage of fluorescence cell intensity of each experimental condition was quantified using Olympus Soft Imaging Solutions. Data were presented as mean ± SD of three or more assays. ^∗∗∗^*p* < 0.001 compared with untreated control cells and ^##^*p* < 0.01 and ^###^*p* < 0.001 compared with UVA-irradiated cells.

The transcription factor AP-1 is a heterodimer protein composed of Fos and Jun subunits that regulate gene expression in response to a variety of extrinsic stimuli through signal transduction mechanisms [27]. The activity of AP-1 subunits from extracellular signals can be modified in certain conditions that are characterized with the balance of keratinocyte proliferation and where differentiation is rapidly and temporally altered [5]. In this study, the effects of EGT pretreatment (0.125-0.5 *μ*M) on the phosphorylation of c-Fos and c-Jun were tested in the presence of exposure to 3 J/cm^2^ UVA. Our Western blot data indicate that only UVA irradiation enhances c-Fos and c-Jun phosphorylation in HSF cells. Nonetheless, EGT pretreatment significantly downregulated this effect (Figure 2(a)). Successively, immunofluorescence staining was done to uncover the nuclear localization of p-c-Fos and p-c-Jun in HSF cells. Pictures showed that UVA-induced nuclear accumulation of p-c-Fos and p-c-Jun was regulated by EGT pretreatment in HSF cells (Figures 2(b) and 2(c)).

### 3.3. UVA-Induced Intracellular ROS Production Was Downregulated by EGT Pretreatment in HSF Cells

**Figure 3:**
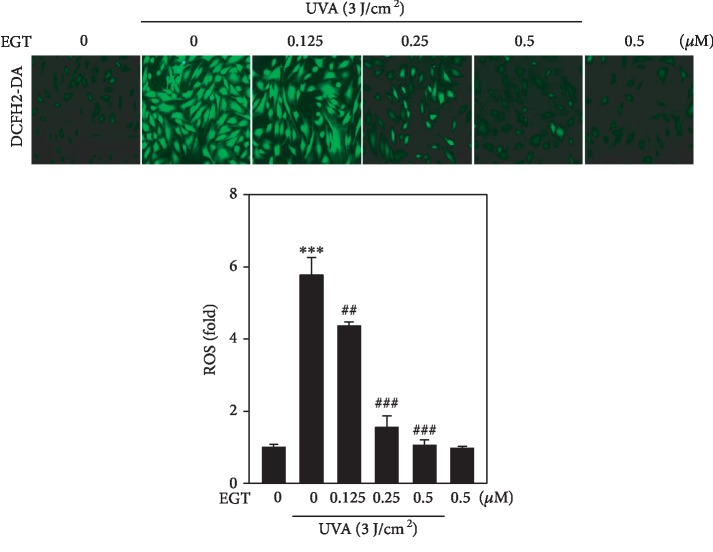
EGT suppressed UVA-induced ROS production in HSF cells. Cells were pretreated with EGT (0.125-0.5 *μ*M) for 24 h and then irradiated with 3 J/cm^2^ UVA. DCF showed intracellular ROS levels and were measured by fluorescence microscopy (200x magnification). 30 min before each experiment was complete, the nonfluorescent, cell membrane-permeable probe DCFH_2_-DA was added to the culture medium with a final concentration of 10 *μ*M. The cells were penetrated by DCFH_2_-DA and then reacted with cellular ROS for metabolization into fluorescent DCF. We quantified the percentage of the fluorescence intensity of the DCF-stained cells with Olympus Soft Imaging Solutions for each condition. Data were presented as mean ± SD of three or more assays. ^∗∗∗^*p* < 0.001 compared with untreated control cells and ^##^*p* < 0.01 and ^###^*p* < 0.001 compared with UVA-irradiated cells.

Pillai et al. reported that excessive production of ROS due to UVA radiation is the principle cause of oxidative damage leading to cancerous condition in skin cells [28]. In this study, using the DCFH_2_-DA fluorescence method, we measured the intracellular ROS levels in UVA-exposed HSF cells.  Figure 3 shows that when compared to the control cells, cells that were not treated with EGT but exposed to UVA irradiation significantly increased the intracellular ROS levels in HSF cells (~5.5-fold). However, EGT pretreatment dose-dependently and significantly downregulated this effect signifying the antioxidant properties exhibited by EGT.

### 3.4. Nuclear Translocation of Nrf2 Was Upregulated due to EGT in HSF Cells

**Figure 4:**
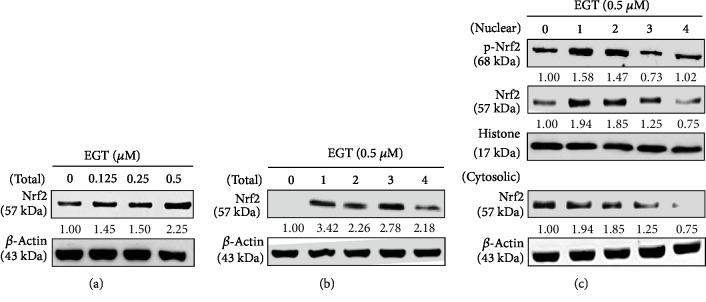
EGT upregulated Nrf2 nuclear translocation in HSF cells. (a, b) EGT upregulated Nrf2 protein levels. HSF cells were treated with different concentrations of EGT (0.125-0.5 *μ*M) for 1 h or 0.5 *μ*M EGT for 1-4 h. Western blot results indicated the effect of EGT on total Nrf2 protein levels in whole cells. *β*-Actin was used as the internal control. Changes in Nrf2 bands were analyzed by densitometry. (c) EGT increased the nuclear translocation of endogenous Nrf2. HSF cells were treated with 0.5 *μ*M EGT for 1-4 h. Western blot results indicated the effect of EGT on Nrf2 protein levels in the nucleus and cytoplasm. For internal controls, histone and *β*-actin proteins were used.

Nrf2, a basic leucine zipper (bZIP) protein, functions as a sensor of oxidative or electrophilic stress and thwarts genome instability. It also adjusts the expression of antioxidant proteins which protects the cells against oxidative damage caused by injury or inflammation [29, 30]. Hence, we hypothesized that EGT could induce the expression of antioxidant genes in UVA-irradiated HSF cells via the Nrf2 pathway. Western blot data demonstrated that EGT dose-dependently increased the expression of total Nrf2 in HSF cells with a maximum expression observed at 0.5 *μ*M of EGT concentration when exposed for 1 h (Figure 4(a)). Using this concentration, we have also tested the effect of time on Nrf2 expression.  Figure 4(b) shows that 0.5 *μ*M of EGT has a biphasic effect on the time. However, maximum Nrf2 expression appeared in the 1 h time point. Later, the effect of time on the expression patterns of activated, nuclear-translocated, and cytosolic Nrf2 levels in the presence of 0.5 *μ*M EGT was tested. Western blot images showed that with the increasing time, cytosolic Nrf2 levels were gradually decreased in the cytoplasm, whereas a maximum nuclear Nrf2 expression (57 kDa) was observed at the 1 h time point that was also immediately converted into p-Nrf2 (68 kDa). This signifies that maximum Nrf2 protein expression was observed within the first hour after 0.5 *μ*M EGT treatment that also facilitated the translocation and phosphorylation of nuclear Nrf2 (Figure 4(c)).

### 3.5. EGT Promoted Antioxidant Protein Expressions and GSH Levels in HSF Cells

**Figure 5:**
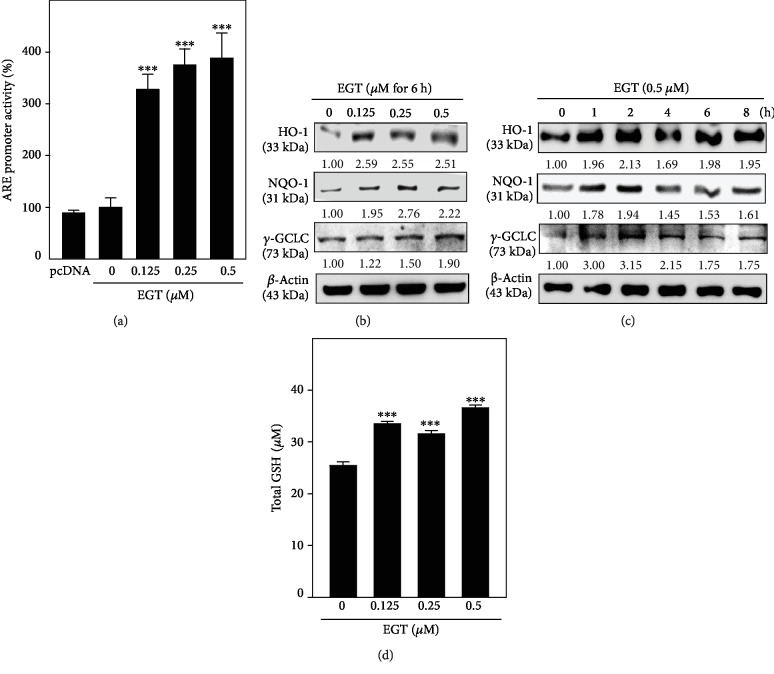
Effect of EGT on ARE promoter activation and subsequent expression of HO-1, NQO-1, and *γ*-GCLC proteins in HSF cells. (a) EGT stimulates Nrf2-mediated ARE activity. HSF cells were cotransfected with pGL3-ARE and treated with different concentrations of EGT (0.125-0.5 *μ*M) for 2 h to measure the percentage of ARE promoter activity. Data were presented as fold over increase in the percentage of ARE promoter activity. (b, c) Effect of EGT concentration and the time of EGT exposure to HSF cells in the induction of antioxidant proteins. HSF cells were treated with different concentrations of EGT for 6 h (b) or 0.5 *μ*M EGT for 1-8 h (c). These cells were harvested, and the expressions of HO-1, NQO-1, and *γ*-GCLC antioxidant proteins were determined by Western blot analysis. In these conditions, *β*-actin was used as an internal control. Relative changes in protein bands were measured by densitometry. (d) EGT upregulated the GSH production. HSF cells were incubated with different concentrations of EGT (0.125-0.5 *μ*M) for 24 h. Intracellular total GSH content was measured by a commercially available ELISA kit, as described in Materials and Methods and was expressed in micromolar concentrations as compared to the untreated cells. Data were presented as mean ± SD of three or more experiments. Statistical significance was considered as ^∗∗∗^*p* < 0.001 as compared to untreated control cells.

The protection against chemically induced oxidative/electrophilic stress is adjusted by a mechanism that uses antioxidant response element- (ARE-) mediated expression and coordinated induction of antioxidant enzymes [31]. HO-1, NQO-1, and *γ*-GCLC are the key antioxidant genes whose enzymes are primarily involved in coping with this stress [30, 32]. Therefore, the ARE-harboring luciferase reporter system was used to demonstrate how EGT could stimulate the transcriptional activity of Nrf2 in HSF cells. Our data has shown that EGT dose-dependently and significantly upregulated the ARE luciferase activity in HSF cells. Nevertheless, luciferase activity of blank plasmid pcDNA in the HSF cells was not changed by EGT (Figure 5(a)). Also, the effect of time and concentrations on EGT-mediated HO-1, NQO-1, and *γ*-GCLC protein expressions showed that EGT concentration has a differential effect on the expression patterns of HO-1 (0.125 *μ*M), NQO-1 (0.25 *μ*M), and *γ*-GCLC (0.5 *μ*M) antioxidant proteins at 6 h (Figure 5(b)), whereas all three proteins exhibited maximum expression at the 2 h time point only when exposed to 0.5 *μ*M EGT (Figure 5(c)).

Glutathione (GSH) is a plentiful nonprotein thiol in the cell for which free radical damage protects them by working as an antioxidant. Inside the cells, glutathione occurs in reduced (GSH) and oxidized (GSSG) states. In healthful cells and tissues, greater than 90% of the total glutathione pool occurs in a reduced form, i.e., GSH, while less than 10% occurs in the GSSG form. GSH is typically found in millimolar concentrations (1-10 mM) [33]. Our ELISA data showed that compared to the control cells, 0.5 *μ*M EGT significantly increased the creation of GSH in HSF cells to signify that EGT is a potent antioxidant inducer and can deal with the oxidative stress posed by UVA irradiation (Figure 5(d)).

### 3.6. EGT Upregulated the Expression of HO-1, NQO-1, and *γ*-GCLC Antioxidant Proteins in HSF Cells via the Nrf2 Pathway

**Figure 6:**
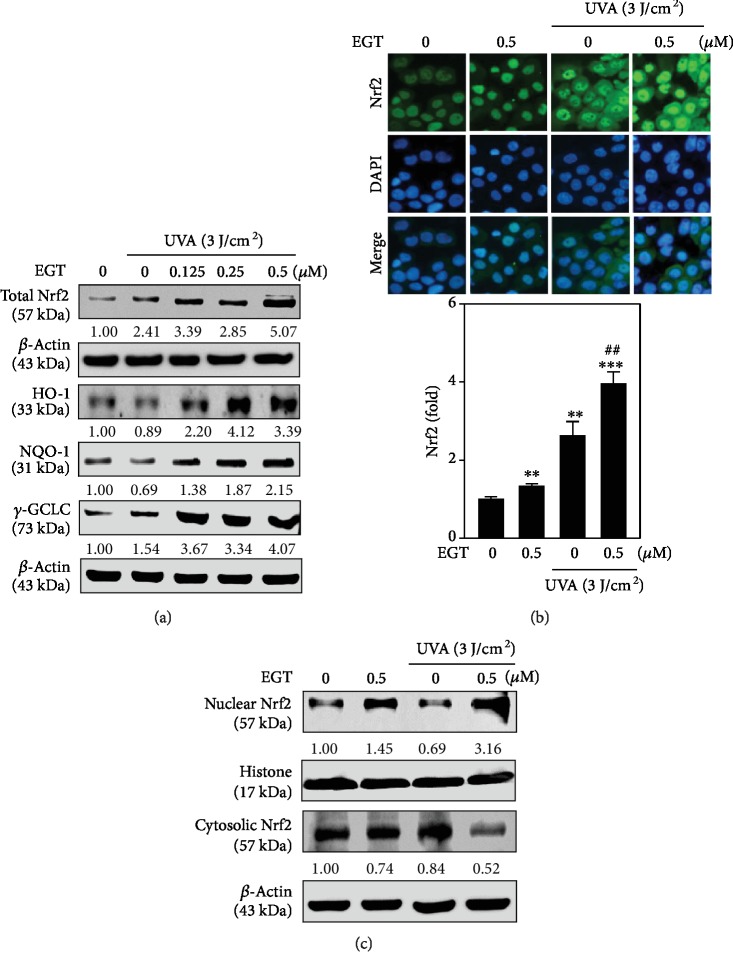
EGT pretreatment facilitated the nuclear translocation of Nrf2 to induce downstream antioxidant protein expression in UVA-irradiated HSF cells. (a) Effect of EGT concentration on total Nrf2, HO-1, *γ*-GCLC, and NQO-1 expression in UVA-irradiated HSF cells. HSF cells were pretreated with EGT (0.125-0.5 *μ*M) for 24 h followed by irradiated without or with 3 J/cm^2^ UVA. Western blot results showing that EGT dose-dependently upregulated total Nrf2, HO-1, *γ*-GCLC, and NQO-1 levels. *β*-Actin as an internal control using densitometry. (b) Immunofluorescence staining of subcellular localization of Nrf2 in EGT-treated and UVA-irradiated cells. HSF cells were pretreated with 0.5 *μ*M EGT for 24 h and then irradiated without or with 3 J/cm^2^ UVA. The percentage of fluorescence cell intensity of each experimental condition was quantified using Olympus Soft Imaging Solutions. (c) Effect of EGT on nuclear translocation of endogenous Nrf2 in UVA-irradiated cells. HSF cells were pretreated with 0.5 *μ*M EGT for 24 h and then irradiated without or with 3 J/cm^2^ UVA. Western blot results showing the effect of EGT on the protein expressions of nuclear as well as the cytosolic Nrf2 levels. Changes in protein expressions were analyzed using densitometry against histone and *β*-actin as the internal controls. Changes in protein expressions were analyzed against *β*-actin as an internal control using densitometry. Data were presented as mean ± SD of three or more assays. ^∗∗^*p* < 0.01 and ^∗∗∗^*p* < 0.001 compared with untreated control cells and ^##^*p* < 0.01 compared with UVA-irradiated cells.

The protective effects of EGT against oxidative stress were assumed to be the result of the induction of antioxidant genes in UVA-irradiated HSF cells. The nuclear activation of Nrf2 is mediated by the induction of antioxidant genes. To underscore this, our experiments on HSF cells were conducted in either the absence or presence of UVA irradiation. First, we tested the validity of EGT concentration on the expression pattern of total Nrf2 in HSF cells. Our Western blot data showed that compared to the control and UVA alone exposed cells, EGT-pretreated and 3 J/cm^2^ UVA-exposed HSF cells showed dose-dependent increase in the expression of total Nrf2 with a maximum expression observed at 0.5 *μ*M EGT concentration (Figure 6(a)). This observation was also consistent with our fluorescence microscopy data (Figure 6(b)). Thus, the expressions of HO-1, NQO-1, and *γ*-GCLC antioxidant proteins in the absence or presence of 3 J/cm^2^ UVA that were pretreated with various concentrations of EGT were measured (Figure 6(a)). Our Western blot data showed that, compared to the control and UVA alone exposed cells, HSF cells exposed 3 J/cm^2^ UVA showed that maximum expression of antioxidant proteins occurred from 0.25 and 0.5 *μ*M concentrations of EGT (Figure 6(a)). Later, we demonstrated the EGT-mediated nuclear translocation of Nrf2.  Figure 6(c) shows that in the presence of 3 J/cm^2^ UVA exposure, EGT pretreatment has facilitated the expression of nuclear Nrf2 with a simultaneous decrease in the cytoplasmic counterpart signifying the nuclear translocation of Nrf2 for the downstream expression of antioxidant proteins. This data suggested that EGT possesses potent antioxidant properties that can help to deal with the UVA irradiation-induced oxidative stress.

### 3.7. EGT-Induced NRf2 Activation Was Mediated by ERK, JNK, and PKC Signaling Pathways in HSF Cells

**Figure 7:**
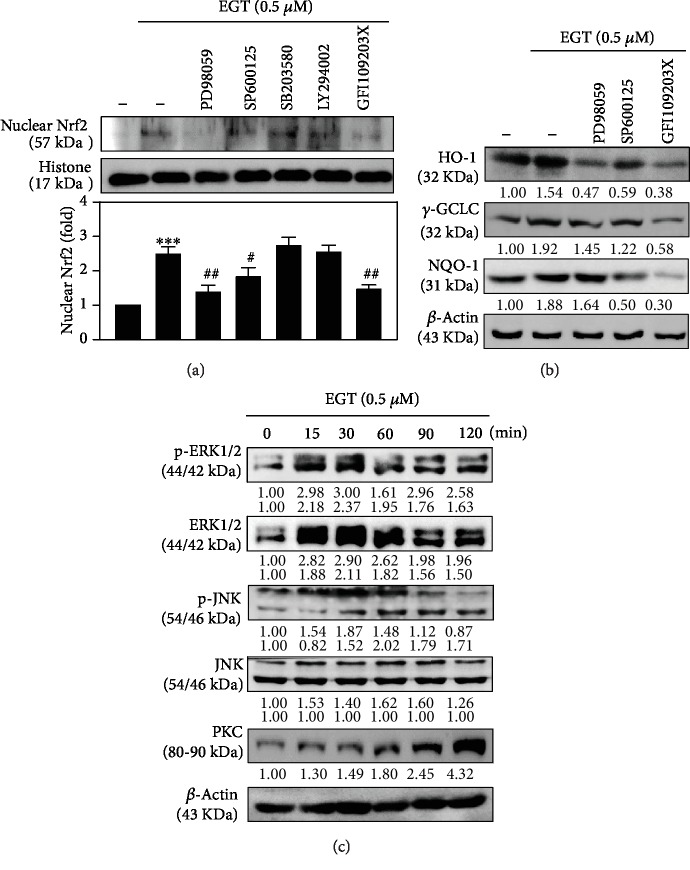
EGT-mediated Nrf2 activation by ERK, JNK, and PKC signaling pathways in HSF cells. (a) Cells were pretreated with pharmacological inhibitors of ERK (PD98059, 30 *μ*M), JNK (SP600125, 25 *μ*M), MAPK p38 (SB203580, 20 *μ*M), PI3K/AKT (LY294002, 30 *μ*M), or PKC (GF109203X, 2.5 *μ*M) for 30 min followed by EGT (0.5 *μ*M) for 1 h. Western blot results showed the nuclear Nrf2 expression with response to inhibitors in the presence of EGT. (b) The protein levels of HO-1, *γ*-GCLC, and NQO-1 were estimated by immunoblot analysis. Cells were pretreated with inhibitors of ERK (PD98059, 30 *μ*M), JNK (SP600125, 25 *μ*M), or PKC (GF109203X, 2.5 *μ*M) for 30 min followed by EGT (0.5 *μ*M) treatment for 6 h. Protein levels of respective markers are significant compared to EGT alone (0.5 *μ*M) treated cells (without inhibitors). (c) EGT activated ERK, JNK, and PKC signaling pathways. Cells treated with 0.5 *μ*M EGT for 15-120 min and the protein levels of activated forms of ERK, JNK, and PKC were evaluated using a specific antibody to p-ERK, ERK, p-JNK, JNK, and PKC by immunoblot analysis. Data were presented as mean ± SD of three or more experiments. Statistical significance was considered as ^∗∗∗^*p* < 0.001 as compared to untreated control cells and ^#^*p* < 0.05 and ^##^*p* < 0.01 as compared to the EGT alone treated cells.

PI3K/AKT, MAP kinase (p38 MAP kinase, ERK, and JNK), and PKC signaling pathways play a part in the activation of Nrf2 [34]. From this, it was hypothesized in this study that one or more of these signaling pathways were implicated in EGT-mediated activation of Nrf2. To demonstrate this, HSF cells were treated with pharmacological inhibitors of ERK (PD98059, 30 *μ*M), JNK (SP600125, 25 *μ*M), p38 MAPK (SB203580, 20 *μ*M), PI3K/AKT (LY294002, 30 *μ*M), or PKC (GF109203X, 2.5 *μ*M) for 30 min followed by 0.5 *μ*M EGT treatment for 1 h. This study's Western blot data indicated that when compared to the control cells, EGT alone treatment significantly upregulated (approximately 1.5-fold) the expression of nuclear Nrf2, whereas only the HSF cells that were cotreated with PD98059 (ERK inhibitor), SP600125 (JNK inhibitor), or GF109203X (PKC inhibitor) along with 0.5 *μ*M EGT significantly downregulated the nuclear NRf2 levels suggesting that only these two pathways were involved in this mechanism (Figure 7(a)). Interestingly, it was observed that compared to the EGT alone treatment, cells cotreated with both EGT and the inhibitors of ERK, JNK, and PKC downregulated the expressions of HO-1, NQO-1, and *γ*-GCLC antioxidant proteins (Figure 7(b)). Later, we demonstrated the effect of time on EGT-mediated activation of these signaling pathways. ERK, JNK, and PKC were activated and showed maximum effect in the order of 30 min, 60 min, and 120 min, respectively, after EGT treatment (Figure 7(c)).

### 3.8. Nrf2 Knockdown Diminishes the Antioxidant Protein Expression in HSF Cells

**Figure 8:**
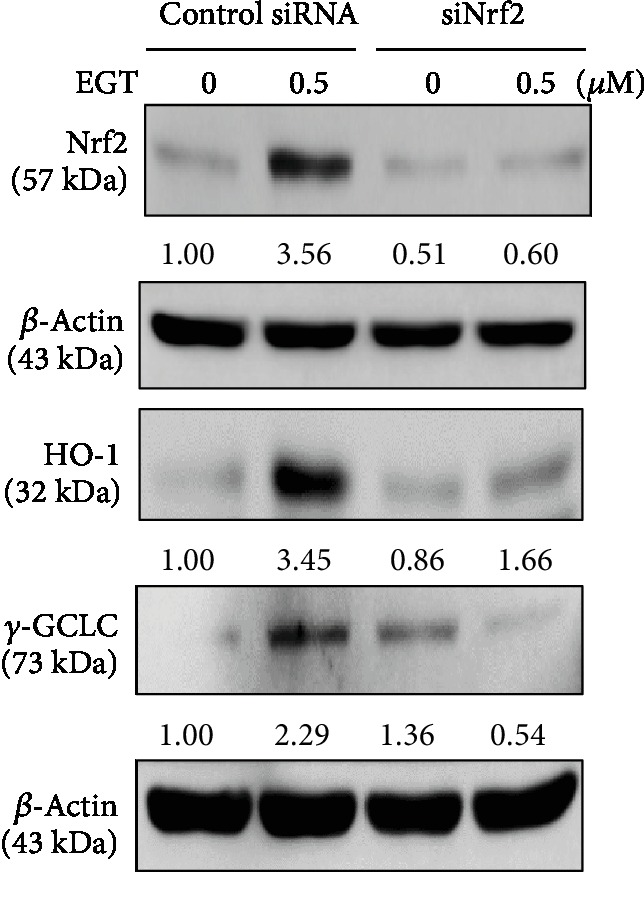
Nrf2 knockdown attenuated the antioxidant protein expression-mediated protective effects of EGT in HSF cells. Cells were transfected with a specific siRNA against Nrf2 or a nonsilencing control. Following the transfection, cells were incubated with EGT (0.5 *μ*M) for 1 or 6 h. The protein levels of Nrf2 (1 h) or HO-1 and *γ*-GCLC (6 h) in control siRNA-transfected and siNRf2-transfected cells were measured by Western blot analysis.

To further emphasize that the activation of Nrf2 is essential for EGT to exhibit dermatoprotective properties, HSF cells were transiently transfected with specific siRNA against Nrf2 or control siRNA, and in the presence or absence of 0.5 *μ*M EGT, antioxidant protein expressions were measured. This study's Western blot data showed that the successful knockdown of Nrf2 was corroborated by blunted Nrf2 levels in siNrf2-transfected cells. Also, there was a remarkable downregulation in HO-1 and *γ*-GCLC protein expression in Nrf2 knockdown cells confirming that Nrf2 plays a significant role in EGT-mediated dermatoprotective properties in HSF cells (Figure 8).

## 4. Discussion

Accumulating evidence suggests that UVA radiation-induced ROS plays a significant role in skin cells and leads to photobiological damage in skin cells. UVA penetrates deep into the skin and damages the dermal compartment leading to wrinkles, photoaging, and even skin cancer [35–37]. EGT is an unusual sulfur-containing amino acid that was discovered a century ago as a constituent in rye ergot [38] and is known to possess antioxidant properties. A unique attribute of EGT has to do with its standard redox potential for the thiol-disulfide couple, which is −0.06 V, and is differentiated from thiols (naturally occurring) and typically range from −0.2 to −0.32 V [20]. HSF cells with submicromolar concentrations of EGT were treated and demonstrated their dermatoprotective efficacy in UVA-irradiated HSF cells. For this, we first tested the effect of EGT (Figure 1(a)) concentration (0.125–0.5 *μ*M) on the viability of HSF cells. Our MTT data suggested that EGT (0.5 *μ*M) did not exhibit any delirious effect on the viability of HSF cells; rather, it has favored cell proliferation (Figure 1(b)). It has been reported that a major collagenolytic enzyme MMP-1 degrades the collagen present in the dermal compartment due to the radiation insult. Therefore, this is considered to be a major enzyme maker in skin cells with respect to the radiation-induced aging [39]. By keeping this view, we tested the effect of EGT on MMP-1 and its substrate collagen protein expressions. Our protein data revealed that EGT dose-dependently upregulated the expression of procollagen levels in HSF cells, whereas cells pretreated with EGT reduced the UVA radiation- (3 J/cm^2^) induced MMP-1 expression confirming that EGT protects the HSF cells from radiation-induced collagen degradation (Figures 1(c) and 1(d)).

Murakami et al. and others have reported that UVA radiation induces the accumulation of intracellular ROS levels in different cell types [40–42]. Our fluorescence data indicated that EGT dose-dependently and significantly downregulated the ROS generation in UVA-irradiated HSF cells contrasted with untreated control and UVA alone treated cells. This data suggested the antioxidant efficacy of EGT (Figure 3). Angel et al. and others have suggested that activation of AP-1, the transcription factor, is important in UVA-induced ROS accumulation in the photoaging process [5, 28]. Protein phosphorylation is a key mechanism involved in the up- and downregulations of activity of transcription factors [43]. Data obtained from the Western blot and immune fluorescence experiments revealed that 0.5 *μ*M EGT pretreatment significantly downregulated the nuclear fractions of p-c-Fos and p-c-Jun proteins. A similar effect was also detected in the nuclear localization of these proteins in UVA-irradiated HSF cells (Figures 2(a)–2(c)). All this data has inferred that EGT pretreatment has inhibited the radiation-induced activation of AP-1 in HSF cells.

Nrf2-ARE is a well-known pathway involved in mediating the expression of various cytoprotective proteins against cellular reactive oxidants and electrophilic stimuli. Activation of this pathway is key for this function. We first measured the activity of Nrf2 followed by ARE promoter activity-mediated antioxidant expression in HSF cells [31]. Nrf2 was partially regulated by its cytosolic protein Keap-1. Nguyen et al. suggested that sequestration of Keap-1 from Nrf2-Keap-1 complex determines the cellular homeostasis [30]. Therefore, we tested the effect of EGT concentration and time on the expression of Nrf2 protein. Western blot data suggested that 0.5 *μ*M EGT has a biphasic time effect on the expression of total Nrf2 (57 kDa) levels. Interestingly, with the increasing time, there was a gradual decrease in the expression of cytosolic Nrf2 levels with a dramatic increase observed in its nuclear counterpart within the first hour after EGT treatment. Also, this nuclear Nrf2 was rapidly undergoing the activation process (p-Nrf2, 68 kDa) (Figures 4(a)–4(c)). Therefore, it was suggestable that EGT activated the dissociation of Nrf2 from cytoplasm to the nucleus that has undergone the activation process for the downstream antioxidant protein expression. By keeping this view, we further measured the ARE promoter activity and its mediated HO-1, NQO-1, and *γ*-GCLC protein expression in HSF cells. Transfection data showed that EGT dose-dependently and significantly upregulated the ARE promoter activity in HSF cells (Figure 5(a)). Also, our Western blot data indicated that EGT concentration has a differential effect on the expression of HO-1 (0.125 *μ*M), NQO-1 (0.25 *μ*M), and *γ*-GCLC (0.5 *μ*M) antioxidant proteins at 6 h (Figure 5(b)). However, in the presence of 0.5 *μ*M EGT, maximum expression of all three proteins was observed within 2 h (Figure 5(c)). EGT increased intracellular GSH levels against UVA-induced reduction in extension lines of evidence (Figure 5(d)). In proportion with the upregulated *γ*-GCLC gene, there are also increased GSH levels and these are responsible for GSH synthesis [33]. To the best our knowledge, this is the first time this result has been reported; i.e., submicromolar concentrations of EGT elevate GSH levels via the upregulated *γ*-GCLC expression and are also mediated by the Nrf2/ARE signaling pathway.

From the above observations, we further tested the effect of EGT pretreatment on UVA-irradiated HSF cells. Figure 6(a) indicates that HSF cells pretreated with EGT followed by irradiation with 3 J/cm^2^ UVA showed dose-dependent increase in the expression of total Nrf2 with a maximum expression observed at 0.5 *μ*M EGT concentration. The same was also evidenced from the fluorescence data (Figure 6(b)). Moreover, EGT pretreatment showed decreased cytosolic and increased nuclear translocation of Nrf2 levels in UVA-irradiated cells that was corroborated with the increased expression of HO-1, NQO-1, and *γ*-GCLC antioxidant proteins (Figure 6(a)) signifying that in the presence of UVA radiation, EGT favors the nuclear translocation of Nrf2 leading to the downstream expression of antioxidant proteins in HSF cells thus confirming the EGT's antioxidant property (Figure 6(c)).

Chen et al. reported that various signaling pathways were engaged in the activation and regulation of Nrf2 [32]. Therefore, we also tested the signaling pathways involved in the activation of Nrf2. Our pharmacological inhibition of various signaling pathway data revealed that in the presence of EGT, only ERK and PKC pathways were mediating the activation and regulation of Nrf2 in HSF cells (Figure 7(a)). One of the interesting factors observed in this context was that ERK, JNK, and PKC were involved in the EGT-mediated expression of all three antioxidant proteins HO-1, NQO-1, and *γ*-GCLC. Nonetheless, when compared to the other pathways, the PKC pathway indicated a more robust effect (Figure 7(b)). Furthermore, the time taken for this effect was also measured. As Figure 7(c) shows, ERK, JNK, and PKC pathways were activated and indicated the maximum expressions at 30, 60, and 120 min, respectively, after 0.5 *μ*M EGT treatment. Further evidence regarding the role of Nrf2 in antioxidant protein expression was also demonstrated using the Nrf2 knockdown studies. There was a remarkable downregulation in the expressions of HO-1 and *γ*-GCLC proteins in Nrf2 knockdown cells confirming that Nrf2 plays a critical role in mediating the EGT's dermatoprotective properties in HSF cells (Figure 8).

## 5. Conclusion

In summary, this study delineated the molecular mechanisms that are fundamental for the EGT-induced Nrf2 activation and its downstream protective effects in UVA-irradiated HSF cells. EGT increased the cell viability and suppressed the UVA-induced expression of collagenolytic MMP-1 leading to the prevention of HSF cells to undergo extracellular matrix damage and degradation. Moreover, EGT favors the nuclear translocation and activation of Nrf2 to mediate the induction of HO-1, NQO-1, and *γ*-GCLC gene expression. Pharmacological inhibition data showed that the ERK, JNK, and PKC pathways were implicated in the activation of Nrf2 and in antioxidant protein expression. However, this effect was inhibited inNrf2 knockdown cells signifying that Nrf2 is crucial for these effects. Our findings from this study suggest that EGT is effective for the treatment of UVA-induced skin damage. Also, EGT should be used in the development of new skin care products or healthier sunscreen products.

## Data Availability

The data used to support the findings of this study are included within the manuscript.
